# Exploring Systemic Comorbidities and Lifestyle Factors Associated With Seborrheic Dermatitis: A Scoping Review

**DOI:** 10.7759/cureus.73555

**Published:** 2024-11-12

**Authors:** Kimberly A Kluglein, Shannon C South, Erjola Toska, Riley Nadolny, Alexis Yagoda, Stephanie J Krusz

**Affiliations:** 1 Dr. Kiran C. Patel College of Osteopathic Medicine, Nova Southeastern University, Clearwater, USA; 2 School of Medicine, Lake Erie College of Osteopathic Medicine, Bradenton, USA; 3 Dr. Kiran C. Patel College of Osteopathic Medicine, Nova Southeastern University, Davie, USA; 4 Department of Internal Medicine, South County Hospital, Warwick, USA

**Keywords:** metabolic syndrome, osteoarthritis, osteoporosis, parkinson' s disease, seborrheic dermatitis

## Abstract

Seborrheic dermatitis (SD), a chronic inflammatory skin condition consisting of itchy, red patches with greasy scales, has been linked to numerous systemic conditions. This review evaluates comorbidities and lifestyle factors that are associated with seborrheic dermatitis. A literary search was conducted in August 2024 using EMBASE, PubMed, and Medline Industries, and evaluated according to Preferred Reporting Items for Systematic Reviews and Meta-Analyses (PRISMA) guidelines. The following results were found from this search. Diet may play a role in seborrheic dermatitis as participants with a diet high in simple carbohydrates, high in vitamin D, and low in iron have a higher instance of disease. Femoral cartilage thickness (FCT), a potential indicator of early osteoarthritis, was greater in patients with SD than in controls and was positively correlated with increasing SD severity. SD has also been associated with chronic diseases. Nearly half of Parkinson’s disease (PD) patients in one study also had SD. As the severity of Parkinson’s disease symptoms increases, the development of SD was observed to occur at a much higher rate, with the burden of SD positively correlated to the severity of PD symptoms. In one cross-sectional study of patients with SD, the most common systemic comorbidities were hypertension and diabetes, however, this finding was not statistically significant. Another study found that obesity was common amongst patients with SD, but this finding was also not significant. Visceral protein levels and height, however, were positively associated with increased SD disease severity. Metabolic syndrome and lower high-density lipoprotein (HDL) and triglycerides have been shown to be associated with more severe SD. Osteoporosis had a higher prevalence and faster disease progression in individuals with comorbid SD than in controls. Around 16% of individuals with psychiatric disorders, such as schizophrenia, bipolar disorder, and major depressive disorder, were found to have SD. HIV is a disease with higher incidence of SD, although the prevalence of comorbid SD may be decreasing as the use of antiretroviral therapy increases. These associations highlight the complex nature of seborrheic dermatitis and underscore the necessity for further research to better understand these relationships and improve disease management.

## Introduction and background

Seborrheic dermatitis (SD) is a prevalent chronic inflammatory skin condition, characterized by the appearance of recurrent erythematous and scaly plaques accompanied by pruritus and induration [1,2]. These lesions typically occur in areas with a high concentration of sebaceous glands, such as the scalp, face, anterior chest, axillae, back, and groin [3]. Although the exact cause of SD remains unclear, its pathogenesis is thought to be multifactorial. Several theories have been proposed including disruptions in the skin microbiota, particularly abnormal immune responses to Malassezia and Demodex species [2]. Other factors such as medications (e.g., dopamine antagonists, immunosuppressants, psoralen plus ultraviolet A, and lithium), hormones, environmental triggers, neurological and psychiatric conditions, and nutritional factors also appear to play a role [2,4]. Furthermore, elevated levels of inflammatory cytokines, including interleukin (IL)-1α, IL-1ß, IL-2, IL-4, IL-6, IL-8, IL-10, IL-12, IL-17, and tumor necrosis factor (TNF)-α, are commonly associated with SD [2].

Globally, the prevalence of SD in adults is approximately 5%, affecting all ethnicities and more common in individuals who are immunocompromised or suffer from neurological disorders [2,5]. SD is a chronic disease and patients often experience long-term courses marked by periods of remission and recurrence. Since no definitive cure exists, treatment strategies focus on managing symptoms with topical therapies and lifestyle adjustments [5]. Given SD's chronic nature and theorized pathogenesis, the potential association of SD with various comorbidities has become an area of relevance.

For instance, comorbidities such as osteoarthritis, osteoporosis, metabolic syndrome, and dietary factors share inflammatory components similar to SD’s pathobiology [2,6]. Chronic inflammation is a feature of several other skin diseases, such as psoriasis and hidradenitis suppurativa, and has been linked to other comorbidities [7]. This raises the question of whether SD, like other inflammatory skin disorders, is associated with a broader range of systemic conditions. As a result, this review seeks to explore the potential comorbidities of SD, including osteoarthritis, Parkinson’s disease, diabetes, hypertension, osteoporosis, metabolic syndrome, psychiatric disorders, HIV, and the influence of lifestyle. By identifying these associations, the review aims to provide a better understanding of SD’s impact on overall health and its possible connections to systemic diseases.

## Review

Methods

Search Strategy and Selection Criteria

EMBASE, Medline Industries, and PubMed databases were used to perform a literature search on August 17, 2024. The Preferred Reporting Items for Systematic Reviews and Meta-Analyses (PRISMA) statement by the Cochrane Collaboration was used. The following criteria were used to determine which articles to include: human patient population, primary study, clinical trial, cohort study, case-control, or cross-sectional study. Included articles were published between January 1, 2014 and August 17, 2024. Exclusion criteria consisted of articles not written in English, review articles, case reports, articles only investigating other cutaneous comorbidities or studies conducted on animals. These criteria were selected to fulfill the objectives of this review.

Key Terms

The key terms used to search for articles were: seborrheic dermatitis, comorbidities, PCOS, polycystic ovary syndrome, insulin resistance, metabolic syndrome, hypertension, Parkinson's disease, obesity, diet, nutrition, acute coronary disease, and osteoarthritis.

Databases were searched using the Boolean operators “AND” and “OR” as follows: (("seborrheic dermatitis") AND ("comorbidities" OR "PCOS" OR "polycystic ovary syndrome" OR "insulin resistance" OR “metabolic syndrome” OR "hypertension" OR "Parkinson’s Disease" OR "obesity" OR "diet" OR "nutrition" OR "acute coronary disease" OR "osteoarthritis")).

Evaluation Process

**Figure 1:**
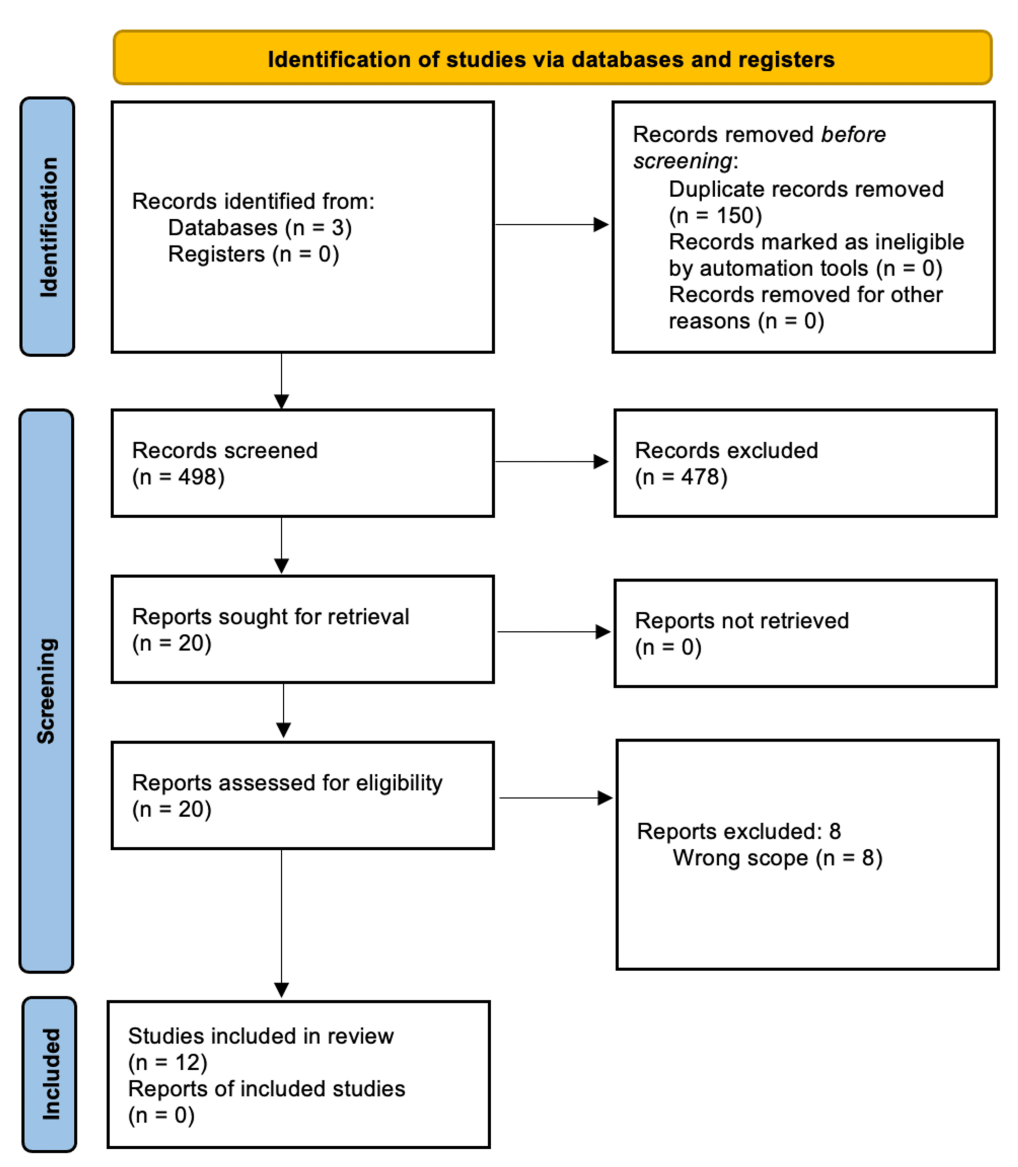
Preferred Reporting Items for Systematic Reviews and Meta-Analyses (PRISMA) diagram

The process of article inclusion is portrayed in the PRISMA diagram (Figure 1). The authors (KK, SS, ET, RN, and AY) screened 498 articles after 150 duplicates were removed. Based on the inclusion and exclusion criteria, 478 articles were excluded and 20 were sought for retrieval as unanimously agreed upon by all five authors. Those 20 articles were obtained and assessed for eligibility. The authors (KK and SS) each independently reviewed the full-text publications. After reviewing all 20 articles, eight were found to be out of the scope of the review. Twelve articles were included in this review. Additional references were utilized in the discussion.

Data Collection

The authors independently evaluated each article and developed a results table, including study reference, study type, sample size, aim, findings, and limitations of the study. The narrative review was written following data entry and analysis of Table 1.

**Table 1:** Study Results ART: anti-retroviral therapy, FCT: femoral cartilage thickness, HDL: high-density lipoprotein, LDL: low-density lipoprotein, LEDD: levodopa equivalent daily dose, LIC: left intercondylar area, LLC: left lateral condyle, LMC: left medial condyle, MRI: magnetic resonance imaging, OA: osteoarthritis, PD: Parkinson's disease, PLWH: people living with HIV, RIC: right intercondylar area, RLC: right lateral condyle, RMC: right medial condyle, SD: seborrheic dermatitis

Reference	Study Type	Sample Size	Aim	Findings	Limitations
Alshaebi M et al. 2023 [1]	Retrospective case-control	267 (59 cases; 208 controls)	To determine what dietary factors are associated with seborrheic dermatitis	A diet high in simple carbohydrates was significantly associated with SD. Frequent consumption of non-acidic fruits, leafy green vegetables, nuts, and coffee was significantly associated with SD. Spicy foods, sweets, fried foods, dairy products, and citrus fruits were frequently associated with disease exacerbations. Prevalence of SD was higher among participants who used butter for frying (p=0.003). Individuals with SD reported a greater intake of visible fat in meat (p=0.049). Consumption of vitamin D was higher among subjects with SD (p=0.014). Among SD patients, a lower proportion of iron consumption was observed (p=0.035).	Small sample size; a predominantly Saudi national cohort may limit generalizability to other populations; subjective, self-reported data
Kulakli S et al. 2024 [2]	Case-control	120 (60 cases; 60 controls)	To determine the relationship between osteoarthritis and seborrheic dermatitis	Pain in the right and left knee was significantly more frequent in patients with SD than controls (p=0.032). No statistically significant differences in morning stiffness, crepitations, or knee widening between 2 groups. FCT was significantly greater in patients with SD than in the control group at all measurement points (RMC, RLC, RIC, LMC, LLC, and LIC) (p<0.05). The FCT value was higher in patients with moderate SD compared with those with mild SD (p<0.001).	Lack of direct radiography or MRI for OA assessment, lack of functional assessments of knee, lack of assessment of other joints like hip or hand, lack of assessment of sex hormones, lack of patients with severe SD
Lu Y et al. 2022 [3]	Population-based cohort study	39,155 (7,831 with SD, 31,324 control)	To investigate whether the existence of SD increases osteoporosis risk by using the Taiwan National Health Insurance Research Database	0.98% of SD patients had osteoporosis. SD group had a 5.95-fold higher osteoporosis risk after adjustment for variables compared to control. Impact of SD on osteoporosis risk was largest in the female and young age groups. In addition, the presence of hyperlipidemia, hyperthyroidism, and epilepsy synergistically increased osteoporosis incidence in the SD group.	Diagnosis of SD and osteoporosis may have been underestimated due to the usage of defined by ICD-9-CM, database lacked detailed data for osteoporosis risk factors
Tomic S et al. 2022 [4]	Primary study	61	To determine the impacts of motor symptoms, nonmotor symptoms, age, sex, and levodopa equivalent daily dose (LEDD) on the appearance and severity of SD in PD patients, and to discuss the possible etiology of SD in PD patients based on the obtained results	SD was present in 32.8% of the 61 patients prior to being diagnosed with PD. SD was correlated positively with the severity of motor symptoms and age, even with adjusting for age, disease duration, and sex. Patients with moderate-to-severe motor symptoms had more severe symptoms. The risk of developing SD was 1.8-fold higher in those with moderate-to-severe symptoms than in those with mild symptoms. No correlation between SD and autonomic dysfunction, sleep disturbances, or other nonmotor symptoms were found. Prior SD did not influence the severity of motor symptoms, the appearance or severity of nonmotor symptoms, or LEDD.	Small sample size
Rajashekar TS et al. 2023 [5]	Cross-sectional study	451	To evaluate the prevalence of SD and associated systemic and cutaneous comorbidities in female patients	There were no statistically significant comorbidities between SD and diabetes, obesity, and hypertension.	Cross-sectional design does not allow for causal inferences; conducted in one center only; the size of the differing age groups of participants varied, the exact number of participants in each group were not listed but appeared to be around 170 participants for the 21-30 age group, 130 for the 31-40 age group, 50 for the 14-20 and 51-60 age groups, 45 participants for the 41-50 age group, and 10 participants for the 61-70 age group
Imamoglu B et al. 2016 [6]	Primary study	83 (47 patients; 36 control)	To investigate metabolic syndrome and to evaluate the relationship between the parameters of the disease and disease severity in patients with SD	A statistically significant inverse correlation between plasma HDL levels and SD severity (p=0.033, r = –0.312), that lower HDL levels may contribute to more severe SD by reducing the regulatory effect on inflammation. No statistically significant between SD severity and other metabolic syndrome components, such as triglycerides, hypertension, abdominal obesity, or fasting glucose	Small sample size; follow-ups were limited
Ozgul A et al. 2023 [7]	Case-control	78 (39 cases; 39 controls)	To examine the relationship between SD and body composition	Positive SD area severity index was seen with height and visceral protein values. No significant findings were found between height, weight, BMI, fat mass, metabolic age, bone density, mineral, visceral adiposity, and protein.	Small sample size; uneven number of men and women in study (for case group 28 vs 12 respectively)
Sanders MGH et al. 2019 [8]	Cross-sectional	4,379 (636 cases; 3,743 controls)	To examine the relationship between dietary patterns and seborrheic dermatitis	Participants with high fruit intake had lower odds of developing SD (p=0.03); those following Western dietary pattern had higher odds of developing SD (p=0.07); no significant association was observed for dietary patterns characterized by high vegetable consumption or fat intake; no significant association was found between SD and total antioxidant capacity	Cross-sectional design does not allow for causal inferences; older cohorts restrict generalizability to younger patients
Shahid W et al. 2020 [9]	Cross-sectional	107	To determine the frequency of the dermatological manifestations associated with PD	High incidence (46.7%) of seborrheic dermatitis in PD patients	Conducted in one center only, limited diversity of the sample, small sample size
Patil S et al. 2022 [10]	Primary study	30	To evaluate the association of lipid profile abnormality and SD by analyzing the lipid profile; and to evaluate the association of lipid profile abnormality and severity of seborrheic dermatitis	A statistical significance between SD severity for serum triglyceride, LDL/HDL ratio, and cholesterol total/HDL ratio, with p values 0.04, 0.03, and 0.004, respectively.	Small sample size
George A et al. 2018 [11]	Hospital-based observational	210	To identify cutaneous manifestations in patients with primary psychiatric disorders	Of the 314 cutaneous manifestations identified in patients with bipolar mood disorder, major depressive disorder, psychosis not otherwise specified, or schizophrenia, SD accounted for 16.2%.	Conducted in one center only, additional comorbidities may confound results
Claasens S et al. 2020 [12]	Cross-sectional, descriptive study	100 patients	To determine the prevalence and spectrum of dermatoses seen in PLWH	The prevalence of mucocutaneous disease in this sample was 12.7%. 6% of participants (PLWH) had seborrheic dermatitis.	Prospective studies conducted in other district-level centers across the country are required to determine the lifetime prevalence and spectrum of dermatoses in PLWH in the ART era

Results

Diet

Alshaebi et al. aimed to explore the relationship between diet and seborrheic dermatitis through a retrospective case-control study involving 267 participants, including 59 diagnosed with SD. Most participants were female (61%), ranging in age from 18 to 30 (36.3%), and predominantly Saudi nationals (96.3%). Data was collected through a self-reported questionnaire and a validated food frequency questionnaire (FFQ) to evaluate dietary intake over the past year. The study revealed a significant association between individuals with a diet high in simple carbohydrates, i.e., white bread, rice, and pasta, and the prevalence of SD. Additionally, daily consumption of leafy green vegetables, non-acidic fruits, nuts, and coffee were also associated with a higher percentage of SD. The type of fat used in cooking was examined; participants who used butter for frying were significantly more likely to have SD. Similarly, individuals with SD reported a greater intake of visible fat in meat. Interestingly, vitamin D intake was significantly higher and iron intake was significantly lower among SD patients compared to those without SD. The study also found that spicy foods, sweets, fried foods, dairy products, and citrus fruits were frequently associated with disease exacerbations [1]. 

Sanders et al. conducted a cross-sectional study to explore the relationship between dietary patterns and incidence of seborrheic dermatitis. The study included 4,379 participants who underwent a full-body skin examination of whom 636 were diagnosed with SD. The median age of the participants was 68.9 years and 57.6% were women. Participants were categorized based on their dietary patterns: the first group followed a vegetable-based diet; the second group adhered to a Western diet characterized by meat, potatoes, and alcohol; the third group followed a fruit-based diet; and the fourth group followed a fat-based diet, which included both healthy and unhealthy fats. It was determined that participants with high fruit intake had lower odds of developing SD, whereas those following a Western dietary pattern had higher odds of developing SD. No significant association was observed for dietary patterns characterized by high vegetable consumption or fat intake. This study also assessed total antioxidant capacity using a ferric-reducing ability of plasma (FRAP) score, however, no association was found between SD and FRAP score [8].

Osteoarthritis

Kulakli et al. performed a case-control study to evaluate the relationship between SD and early-stage osteoarthritis (OA). Sixty patients with mild to severe SD, according to the Seborrheic Dermatitis Area and Severity Index (SDASI) score, were identified and compared to 60 age and sex-matched controls. Knee pain, morning stiffness, crepitation, and widening of the knee joint were evaluated, as well as distal femoral cartilage thickness (FCT) using ultrasound (US) at the central regions of the right medial condyle (RMC), right lateral condyle (RLC), right intercondylar area (RIC), left medial condyle (LMC), left lateral condyle (LLC), and left intercondylar area (LIC) [2]. The researchers found that compared to controls, patients with SD experienced more subjective right and left knee pain, which was a significant finding. Patients in this study did not have appreciable differences in height, weight, BMI, smoking status, exercise habits, or occupation. Additionally, FCT was significantly greater in patients with SD than in the control group at all anatomical points assessed. There was no significant difference in morning stiffness, knee crepitations, or joint widening in patients with SD versus controls. Statistical extrapolation revealed a strong positive correlation between disease severity and FCT at five of the six locations and a moderately positive correlation at LLC, however, there were no patients in this study that had an SDASI score indicating severe SD [2].

Parkinson's Disease

Shadid et al. performed a cross-sectional study of 107 patients with Parkinson's Disease in Pakistan to investigate common dermatological manifestations associated with PD. They found that 46.7% of PD patients also had SD [9]. Another study explored the relationship of motor and non-motor symptoms of PD and the development of SD. Tomic et al. sought to determine the impact of motor symptoms, non-motor symptoms, age, sex, and levodopa equivalent daily dose (LEDD) on the appearance and severity of SD in PD patients. They performed a cross-sectional study on 61 patients with PD, using the Unified Parkinson’s Disease Rating Scale part III to evaluate for motor symptoms. Non-motor symptoms were evaluated using scales for sleep disturbances, autonomic dysfunction, and a non-motor symptom questionnaire. The researchers did not find that prior SD influenced the severity or manifestation of motor or non-motor symptoms, or LEDD. Of the 61 patients, 36.1% were found to have SD. A positive correlation between motor symptoms severity and presence of SD was also found. When utilizing the SDASI score for symptom severity, moderate-to-severe motor symptoms were associated with more severe symptoms of SD. Severe motor symptoms were also associated with an increased risk of developing SD (1.8x higher) than compared to patients with mild motor symptoms [4].

Diabetes and Hypertension

In the cross-sectional study by Rajashekar et al., the relationship between SD and systemic and cutaneous comorbidities was evaluated. The study involved 451 female participants, aged 18 to 70, all diagnosed with SD in Kolar. Pregnant women were excluded from the study. More than half of the participants belonged to the 21-30 and 31-40 age groups. An in-depth history and physical exam were obtained from the participants. The most common systemic comorbidities amongst the participants with SD were diabetes, obesity, and hypertension, however, no statistically significant associations were found between these systemic comorbidities and SD [5]. 

Body Composition

In a case-control study by Ozgul et al., body composition was assessed using bioelectrical impedance analysis. The case group consisted of 39 patients with SD (12 female and 27 male) with a mean age of 25.5 and 39 matched controls (10 females and 29 males) with a mean age of 26.8. The study found no significant differences between the groups in body composition parameters such as fat percentage, muscle mass, total body water, and visceral protein. However, a positive correlation was observed between SDASI and both visceral protein levels and height [7]. In another study, obesity was a common comorbidity seen amongst the participants with SD but the association was not statistically significant [5].

Metabolic Syndrome and Lipid Profile

One study assessed the relationship between SD and metabolic syndrome by comparing 47 SD patients, diagnosed based on clinical features such as erythema, dandruff, and pruritus, with 36 healthy individuals [6]. There was no significant difference found between the groups’ age distribution or gender. The severity of SD was assessed using the SDASI. Both groups were evaluated for metabolic syndrome using Adult Treatment Panel (ATP) III criteria, which included measurements of waist circumference, blood pressure, fasting glucose, triglycerides (TG), total cholesterol, high-density lipoprotein (HDL), low-density lipoprotein (LDL), and high-sensitivity C-reactive protein (hsCRP) to assess the relationship between SD and metabolic syndrome. The study found a statistically significant negative correlation between plasma HDL levels and SD severity. However, no statistically significant associations were found between SD severity and other metabolic syndrome components, such as triglycerides, hypertension, abdominal obesity, or fasting glucose [6].

Another study investigated the relationship between abnormal lipid profiles and the severity (mild, moderate, or severe) of SD in patients [10]. This prospective observational study involved 30 patients with SD, of which 60% were male and 70% were in the 19-30 age group. Among these patients, 30% were classified as having moderate SD and 36.6% as having severe. A lipid profile was assessed for each participant, with results categorized as normal or abnormal for each component: cholesterol, triglycerides, LDL, and HDL [10]. The study, using ANOVA, found a positive correlation between the severity of SD and serum triglyceride levels, LDL/HDL ratio, and cholesterol total/HDL ratio, with p values of 0.04, 0.03, and 0.004, respectively [10].

Osteoporosis

A study utilizing data from the Taiwan National Health Insurance Research Database (NHIRD) assessed whether SD increases the risk of developing osteoporosis. The study included 7,831 patients with SD between the ages of 18-50 and 31,324 matched controls without SD [3]. The controls were matched based on gender, age, index date, and Charlson Comorbidity Index (CCI) at a ratio of 1:4. The mean age of the SD group was 33.1 years, while the mean age of the control group was 33.3 years. The researchers evaluated incidence rates and hazard ratios for osteoporosis in both groups. The incidence of osteoporosis was significantly higher in the SD group (0.98%, n = 77) compared to the control group (0.66%, n = 206) [3]. Furthermore, SD patients developed osteoporosis at a much faster rate, with onset occurring 2.2 years after enrollment compared to 8.9 years in the control group. The study also found that the risk of osteoporosis was 5.95 times higher in the SD group than in the control group. The incidence rate of osteoporosis increased with age in all SD patients, however, the risk was disproportionately higher among those in their third decade of life. SD patients within the age group of 30-39 had a higher osteoporosis risk compared to all other age groups [3].

Psychiatric Disorders

George et al. conducted a hospital-based observational study to identify cutaneous manifestations in patients with primary psychiatric disorders. The study recruited 210 patients from outpatient and inpatient departments of Justice K S Hegde Charitable Hospital, Deralakatte, Mangalore. The majority of participants were female (58.1%) and in their third to fifth decades of life. A psychiatrist initially assessed the patients for primary psychiatric disorders, after which a dermatologist evaluated the associated skin conditions. The most prevalent psychiatric disorders observed were schizophrenia, major depressive disorder, bipolar mood disorder, and psychosis not otherwise specified. Among the 314 cutaneous manifestations identified in these patients, seborrheic dermatitis was most frequently observed, accounting for 16.2% across all psychiatric disorders. However, the authors did not report a statistically significant association of any noninfective dermatitis [11].

HIV

One study by Claasens et al. aimed to determine the prevalence and manifestations of dermatoses in individuals with HIV. A cross-sectional, descriptive study conducted at Karl Bremer Hospital, a district-level facility in the Western Cape province of South Africa, included 970 people living with HIV (PLWH). Out of the 970 participants, 59% were female, 75% were black, 69% had CD4+ cell counts over 200, and 74% were taking antiretroviral therapy. The mean age of the participants was 40.4 years [12]. The study found that 6% of participants had SD, making it the second most common inflammatory dermatosis in the study. There were no significant associations between skin manifestations (infectious, such as fungal dermatoses, and non-infectious, such as SD), and demographics (gender, ethnicity, and BMI) or severity of HIV (CD4+ count, duration of antiretroviral therapy, and viral load) [12].

Discussion

This review aimed to identify the comorbidities associated with seborrheic dermatitis, a prevalent papulosquamous skin condition affecting approximately 5% of the global population [13]. Our analysis identified several significant associations between seborrheic dermatitis and various comorbid conditions, such as osteoarthritis, Parkinson’s disease, metabolic syndrome, and osteoporosis. 

The potential relationship between SD and each of the comorbidities is not fully understood. Theories suggest that elevated levels of insulin-like growth factor 1 (IGF-1) and androgens may stimulate increased sebum production. This pathogenesis is associated with simple carbohydrate diets [1] and obesity [14], respectively. Diabetes is also known to activate IGF-1 receptors and stimulate androgen production through several mechanisms, including hyperinsulinemia [15]. Ozgul et al. also theorized that elevated IGF-1 levels were associated with SD, based on a positive correlation found between SDASI scores and height, although this correlation did not align with prior literature. Regarding diet, the study by Alshaebi et al. revealed an association between diets high in simple carbohydrates, leafy green vegetables, non-acidic fruits, nuts, and coffee with the prevalence of SD. In contrast, Sanders et al. found that high fruit intake was associated with lower odds of developing SD, while adherence to a Western dietary pattern was linked to higher odds. The conflicting results regarding fruit intake and its effect on SD may be influenced by factors such as the type of fruit consumed, the quantity, preparation methods, and other dietary components.

One theory focuses on the relationship between visceral fat levels and SD. Elevated levels of visceral fat can lead to increased production of free fatty acids. These free fatty acids can trigger gluconeogenesis and the production of insulin, triglycerides, and very low-density lipoproteins (VLDL) in the liver, creating a cycle where higher insulin levels further drive free fatty acid production. This cycle can contribute to the development of diabetes, while also causing inflammation that may trigger SD [16]. Excessive visceral adipose tissue has also been proposed to release inflammatory cytokines, such as TNF-α, IL-6, leptin, and resistin, leading to chronic inflammation. This inflammation can activate the renin-angiotensin-aldosterone system, contributing to both hypertension and SD [16]. Metabolic syndrome, which is closely related to increased visceral fat and diabetes, shares a common foundation of inflammation. It involves inflammatory cytokines such as C-reactive protein (CRP), IL-6, and TNF-α, as components of its pathogenesis [6]. The study by Imamoglu et al. found a statistically significant inverse correlation between plasma HDL levels and SD severity, suggesting that lower HDL levels may contribute to more severe SD by reducing the regulatory effect on inflammation [6]. 

Another theory is that SD may be triggered by chronic inflammation and pro-inflammatory cytokines. Obesity has been hypothesized to be associated with chronic inflammation, particularly in conditions like metabolic syndrome, through inflammatory cytokines such as CRP, IL-6, and TNF-α [17]. Additionally, dietary factors may impact antioxidant levels and metabolic profiles, potentially contributing to the development or exacerbation of SD [1]. Osteoporosis and OA share common pathobiological mechanisms with SD [2,3]. Inflammatory cytokines such as IL-1ß, IL-6, IL-17, and TNF-α are prevalent in both OA and SD [2]. The study by Kulakli et al. found that young patients with SD had an increased FCT, which could be associated with early-stage osteoarthritis. This highlights the question of whether the severity of SD influences the likelihood of developing OA.

Seborrheic dermatitis is also proposed to be associated with chronic diseases, such as Parkinson’s disease and HIV, and may be exacerbated by psychological stress. SD has been observed as a common cutaneous manifestation of psychotic disorders [11]. In Parkinson’s disease, SD presents through various proposed mechanisms, including increased sebum production and an impaired immune response to Malassezia spp. [9], however, there is no definitive answer for its etiology [4]. This is similar to the theory that yeast associated with SD might trigger an inflammatory response, stimulating cytokine production and activating Ang-II type I receptors, potentially leading to hypertension [17]. Regarding the relationship between PD and SD, Tomic et al. suggested that the severity of motor symptoms influences the presence of SD, rather than SD affecting motor symptoms. This was supported by a positive correlation between the severity of motor symptoms and the presence of SD.

Dermatological manifestations, including SD, are well-documented in PLWH. Historically, higher prevalence rates of SD have been reported among HIV-positive patients, ranging from 40% to as high as 80% in those with AIDS, compared to 3% in HIV-negative individuals [12]. However, more recent studies have shown that with the widespread use of anti-retroviral therapy (ART), prevalence rates are lower. This aligns with the lower prevalence of SD observed in the study by Claasens et al. and suggests that ART may be reducing the frequency or severity of SD among PLWH. This underscores the need for ongoing research to better understand the impact of ART on the prevalence of SD and its implications for clinical management [12].

For many of these conditions, individuals often have other associated comorbidities that can complicate the direct pathophysiology of the relationship with SD. For instance, when investigating the relationship between obesity and SD, it’s important to consider that obesity frequently coexists with other conditions such as diabetes, insulin resistance, metabolic syndrome, cardiovascular disease, hypertension, and poor dietary habits-all of which may influence the onset or severity of SD. These associations highlight the complex nature of SD and underscore the necessity for further research to better understand these relationships and improve disease management. 

Recognizing the associations between SD and comorbid conditions can facilitate early identification of at-risk populations and guide the development of new treatments. By addressing these associated conditions, individuals may significantly lower their chances of developing seborrheic dermatitis. Furthermore, the comorbidities highlighted in this review emphasize the importance of comprehensive patient evaluations. Clinicians should not only focus on treating the cutaneous symptoms of SD but also on identifying and managing underlying systemic conditions that may exacerbate the disease. Adopting treatment strategies that target both SD and its associated conditions may lead to improved patient outcomes and a better quality of life.

While this review offers promising insights into the associations between comorbidities and SD, several limitations exist. Many of the studies reviewed focused on specific populations, such as Saudi nationals, which may limit the generalizability of the findings. Future research should involve larger and more diverse cohorts to enhance the applicability of the results. Additionally, variables such as environmental factors, genetic predisposition, and other systemic or cutaneous conditions could influence susceptibility to SD and potentially confound the findings. 

## Conclusions

This review highlights the association between seborrheic dermatitis and various comorbid conditions such as osteoarthritis, Parkinson’s disease, diabetes, hypertension, metabolic syndrome, osteoporosis, primary psychiatric disorders, and HIV. Factors such as body composition and diet were evaluated to see if they influence the prevalence of seborrheic dermatitis. While patients may initially present with only skin-related symptoms, healthcare providers should be aware of potential underlying systemic issues. Screening and recognition of these associated conditions may lead to earlier diagnosis and better management, improving the patient’s quality of life. More research should be done to better understand the connection between seborrheic dermatitis and these comorbid conditions.
